# Deceptive single-locus taxonomy and phylogeography: *Wolbachia*-associated divergence in mitochondrial DNA is not reflected in morphology and nuclear markers in a butterfly species

**DOI:** 10.1002/ece3.886

**Published:** 2013-11-25

**Authors:** Ullasa Kodandaramaiah, Thomas J Simonsen, Sean Bromilow, Niklas Wahlberg, Felix Sperling

**Affiliations:** 1 School of Biology, Indian Institute of Science Education and Research Thiruvananthapuram, CET College campusThiruvananthapuram, 695016, India; 2 Department of Zoology, Stockholm University106 91, Stockholm, Sweden; 3 Department of Life Sciences, Natural History MuseumCromwell Road, London, SW7 5BD, U.K; 4 Department of Biological Sciences, University of AlbertaEdmonton, AB T6G 2E9, Canada; 5 Laboratory of Genetics, Department of Biology, University of Turku20014, Turku, Finland

**Keywords:** *Coenonympha california*, *Coenonympha nipisiquit*, *Coenonympha tullia*, cryptic diversity, incipient species, phylogeography, *Wolbachia*.

## Abstract

The satyrine butterfly *Coenonympha tullia* (Nymphalidae: Satyrinae) displays a deep split between two mitochondrial clades, one restricted to northern Alberta, Canada, and the other found throughout Alberta and across North America. We confirm this deep divide and test hypotheses explaining its phylogeographic structure. Neither genitalia morphology nor nuclear gene sequence supports cryptic species as an explanation, instead indicating differences between nuclear and mitochondrial genome histories. Sex-biased dispersal is unlikely to cause such mito-nuclear differences; however, selective sweeps by reproductive parasites could have led to this conflict. About half of the tested samples were infected by *Wolbachia* bacteria. Using multilocus strain typing for three *Wolbachia* genes, we show that the divergent mitochondrial clades are associated with two different *Wolbachia* strains, supporting the hypothesis that the mito-nuclear differences resulted from selection on the mitochondrial genome due to selective sweeps by *Wolbachia* strains.

## Introduction

Molecular data are being increasingly preferred over morphological traits for species identification and discovery – the exponentially growing number of DNA barcoding sequences without species names stands testimony to this (Parr et al. 2012; http://iphylo.blogspot.com/2011/04/dark-taxa-genbank-in-post-taxonomic.html). Mitochondrial DNA (mtDNA) has been especially popular, becoming the marker of choice in numerous phylogeographic, population genetic, and molecular taxonomic studies. However, mitochondrial history does not always reflect the true history of species being studied and is often incongruent with nuclear data (Dupuis et al. 2012).

Differences between the observed geographical patterns of mitochondrial and nuclear gene variation may be caused by introgression. Mitochondrial genes are less constrained by linkage to selected loci and hence are expected to introgress deeper than their nuclear counterparts (reviewed in Harrison 1990; Funk and Omland 2003). Mitochondrial introgression has commonly been reported in Lepidoptera (e.g., Sperling 1993; Wahlberg and Nylin 2003; Gompert et al. 2006; Salazar et al. 2008), despite the prediction, on the basis of Haldane's rule, that for groups like Lepidoptera in which females are the heterogametic sex, introgression of maternally inherited mtDNA should be unlikely to happen (Sperling 2003).

Incomplete lineage sorting can also lead to pronounced differences between mtDNA and nDNA. Following the formation of two daughter species from an ancestor, lineage sorting is more rapid in the case of mtDNA due to its lower effective population size (Funk and Omland 2003; Hudson and Turelli 2003). Therefore, incomplete lineage sorting in young species (either of nDNA or both) can potentially result in differences in population structures inferred from the two kinds of markers. Sex-biased dispersal may also lead to such differences (Galtier et al. 2009).

Another potential cause of mito-nuclear discordance is selection on the mitochondrial genome, for instance, due to cytoplasmic parasites. In particular, bacteria in the genus *Wolbachia* are widespread cytoplasmic parasites that induce a variety of phenotypic effects – male killing, cytoplasmic incompatibility (where males infected with a *Wolbachia* strain are reproductively incompatible with females that are not infected by the same strain), and feminization (where genetic males develop into functional females) – that assist rapid spread of the bacterium in host populations (Werren 1997). Mitochondrial haplotypes can, and will, hitchhike along with the bacteria, leading to drastic changes in population structure of the mitochondrial genome (Johnstone and Hurst 1996; Turelli and Hoffmann 1996; Hurst and Jiggins 2005; Charlat et al. 2009). Furthermore, a rare introgression event that is accompanied by *Wolbachia* can result in the introgressed mitotype being fixed rapidly (Jiggins 2003; Hurst and Jiggins 2005).

Despite a large amount of literature discussing these problems, mtDNA remains the first choice for phylogeographic and barcoding studies within animals. Although nuclear data such as microsatellites and RAD tags are potentially much more variable and informative at the population genetic level, mtDNA is more suited for inferring gene-level phylogeographic history due to low or absent recombination and relatively fast substitution rates. We here illustrate why reliance solely on mitochondrial markers can lead to highly misleading phylogeographic interpretation and barcoding analyses.

Our study builds on Bromilow and Sperling (2011) who, in a project focused on conservation genetics of grassland butterflies in the threatened Peace River region of Northern Alberta, found surprisingly deep mtDNA divergence within populations of *Coenonympha tullia* (Müller, 1764). One mtDNA lineage was sister to all other North American haplotypes, yet this divergent lineage occurred only in a previously glaciated region of northern Alberta. Although this butterfly species was previously thought to be widespread across temperate regions of North America, Europe, and Asia (Bozano 2002), recent work has shown that North American “*C. tullia*” populations are phylogenetically distinct from European *C. tullia* (Kodandaramaiah and Wahlberg 2009) and should be considered a separate species. The taxonomic changes involved are beyond the scope of this article and we thus refer to the North American taxon as “*C. tullia*.” Within North America, the species is distributed from the Canadian Maritimes to the Pacific coast from Alaska to California but absent from southeastern USA (e.g., Scott 1986; Pelham 2008), with a large number of more or less tenable species level taxa (Pelham 2008). In North America, the butterfly feeds on grasses during larval stages and occurs in grasslands, parkland, and mixed forest habitats (Scott 1986; Bird et al. 1995; Layberry et al. 1998; Sei and Porter 2003, 2007).

To better understand the causes of the anomalous mtDNA divergence within “*C. tullia*”, we first examined variation in male genitalic morphology and a nuclear gene to test whether the mtDNA differences corresponded to variation in the rest of the genome. Such correspondence would indicate cryptic species, but this pattern was not supported in our results. We then tested for nonrandom associations between mtDNA and the presence of strains of *Wolbachia*, which are endosymbionts that occur in up to half of all nymphalid butterflies (E. Hornett, N. Wahlberg & U. Kodandaramaiah, unpubl. data).

## Methods

### Sampling and DNA extraction

**Figure 1:**
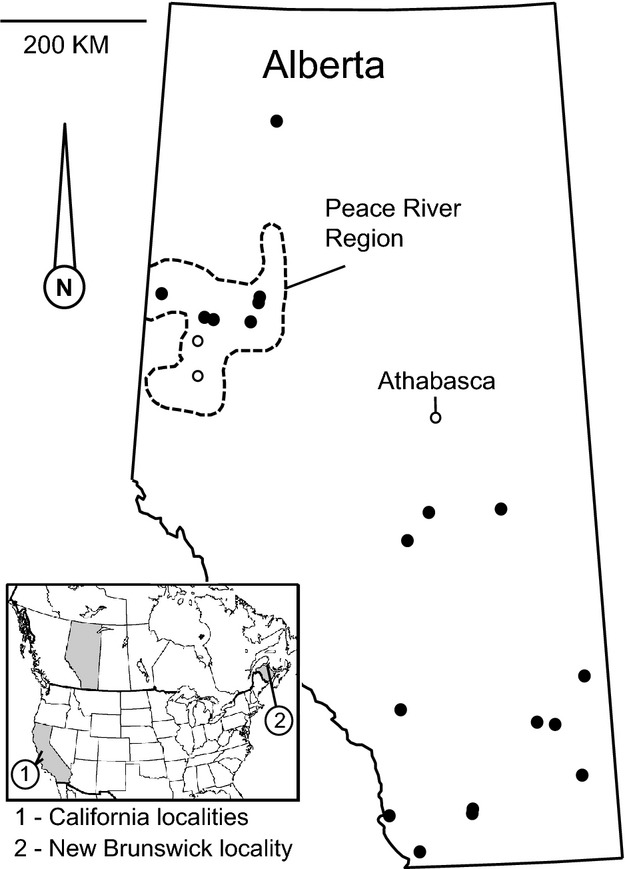
Collection localities of individuals with COI sequence data used in the study. Empty circles indicate localities where the divergent *C. tullia* mitotype was found. The inset map shows the relative locations of the three collecting regions (California, USA; Alberta, Canada; and New Brunswick, Canada).

Individuals from 27 localities across Alberta, together with one locality from New Brunswick and two from California, were used in this study (Fig. 1), for a total of 49 specimens. Samples were collected by authors and collaborators between 1996 and 2006, and DNA was preserved by storing two legs in ethanol. DNA was extracted using DNeasy Blood and Tissue Kit (Qiagen, Hilden, Germany).

### Sequencing COI and RpS5

New COI sequences were generated for this study in addition to those used in Bromilow and Sperling (2011). COI was amplified with two primer pairs LCO (5′ G GTCAACAAATCATAA AGATATTGG 3′) – HCO (5′ TAAACTTCAGGGTGACCAAAAAATCA 3′) and Jerry (5′ CAACAYTTATTTTGATTTTTTGG 3′) – Pat (5′ ATCCATTACATATAATCTGCCATA 3′) with the universal forward (T7 promoter: TAA TAC GAC TCA CTA TAG GG) and reverse (T3: ATT AAC CCT CAC TAA AG) tails concatenated (see Wahlberg and Wheat 2008). The universal tails improve amplification yields and allow sequencing of various PCR products to be carried out with the tails. PCR conditions were as follows: (1) 95°C for 5 min (2) 40 cycles of 94°C for 30 sec, 50°C for 30 sec, 72°C for 1 min, and (3) a final extension period of 72°C for 10 min. Purified PCR products were sequenced with both the forward and reverse tails by a commercial sequencing company (Macrogen Inc., Seoul, South Korea). Chromatograms were visualized, and sequences were aligned by eye in Bioedit, version 7.2.2 (Hall 1999). The primers RpS5degF (TAATACGACTCACTATAGGGATGGCNGARGARAAYTGGAAYGA) and RpS5degR (ATTAACCCTCACTAAAGCGGTTRGAYTTRGCAACACG; Wahlberg and Wheat 2008) along with respective universal tails were used to amplify RpS5. PCR and sequencing protocols were the same as for COI, except that the annealing temperature was 55°C.

A total of 1487 bp of COI sequence from 49 North American “*tullia*” samples representing 30 localities were used in the study (Figs 1, S1). RpS5 was sequenced for a total of 33 samples (Fig. S1), of which 30 also had COI data.

### Phylogenetic reconstructions

Maximum likelihood analyses were conducted in Phyml 3.0 (Guindon and Gascuel 2003; Guindon et al. 2010) using the SeaView, version 4.3.2 (Gouy et al. 2010) interface. The best fitting model for each dataset was estimated based on the Bayesian Information Criterion using jModeltest, version 1.1 (Posada 2008) in conjunction with Phyml. The best fitting models were as follows: COI−HKY + I; RpS5−K80 + I; combined dataset of *Wolbachia* genes – HKY). Heuristic searches were performed using both NNI (nearest neighbour interchange) and SPR (subtree pruning regrafting), with the transition/transversion ratio optimized. Bootstrap support values were estimated based on 1000 pseudorandom replicates. Before analyses on the North American “*tullia*” samples, we first performed a maximum likelihood phylogenetic analysis to confirm that these individuals were monophyletic with respect to other *Coenonympha* species and *C. tullia* from Europe. The COI gene sequences from Kodandaramaiah and Wahlberg 2009 were analyzed along with the North American “*tullia*” sequences and five newly sequenced *tullia* samples from Russia. A *Wolbachia* strain from the nymphalid butterfly *Polygonia c-album* (Kodandaramaiah et al. 2011) was used as an outgroup for the phylogenetic analysis of *Wolbachia* sequences.

### Measures of genetic diversity and differentiation

Ф_ST_, haplotype diversity and nucleotide diversity values were calculated in Arlequin 3.2 (Excoffier et al. 2005). Statistical significance of Ф_ST_ value between Clade I and II was tested based on 10,000 permutations. Mean percent sequence divergence between clades was calculated in MEGA, version 5.05 (Tamura et al. 2011) using the ‘pairwise deletion’ option, but with other settings at their default values.

### Haplotype determination

As most sequences in the diploid RpS5 dataset contained heterozygotes, we were unable to manually identify the haplotype phase for each individual. We therefore employed a coalescent-based Bayesian approach implemented in the program PHASE, version 2.0 (Stephens et al. 2001; Stephens and Donnelly 2003). The PHASE algorithms were invoked and run through the software DNAsP, version 5.10.01(Librado and Rozas 2009), with default settings. Although not as accurate as cloning and sequencing of amplicons, such computational methods have been shown to be reliable enough to be used in phylogeographic and population genetic studies based on nuclear sequence data (Harrigan et al. 2008).

### *Wolbachia* assays

wsp was amplified using wsp81F (5′-TGG TCC AAT AAG TGA TGA AGA AAC3-′) and wsp691R (5′-AAA AAT TAA ACG CTA CTC CA-3′; Zhou et al. 1998), with the universal tails attached. PCR protocols were the same as for RpS5, and all reaction sets included positive and negative controls (dH_2_O). Success of amplification was tested using 8 microliters of PCR product run in a standard 1% agarose gel electrophoresis with ethidium bromide staining. gatB and ftsZ were amplified using the primer pairs gatB_R1: TGGYAAYTCRGGYAAAGATGA – gatB_F1: GAKTTAAAYCGYGCAGGBGTT and ftsZ_F1 ATYATGGARCATATAAARGATAG – ftsZ_R1: TCRAGYAATGGATTRGATA (Baldo et al. 2006), all of which had the universal tails. PCR protocols used for COI and RpS5 were followed, but with annealing temperature set at 54°C. The three *Wolbachia* genes were sequenced from a total of 24 samples. Data from all genes were obtained from 16 samples, six samples lacked data from one gene, while two samples had sequences only from the wsp gene.

### Morphological examinations

**Figure 2:**
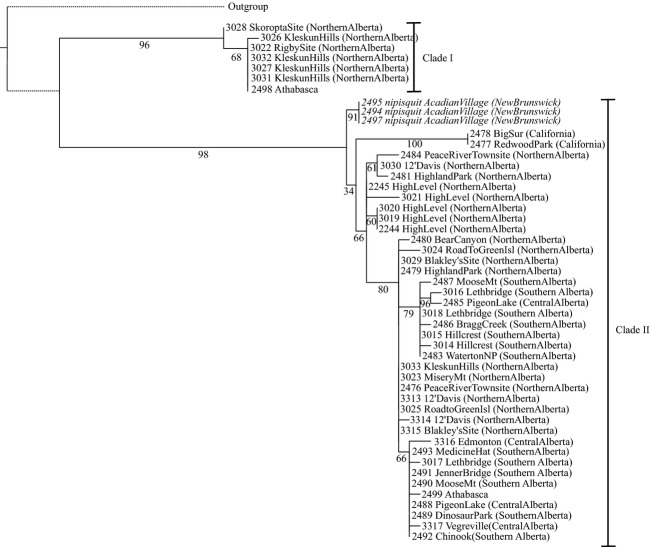
Maximum likelihood phylogeny of the mtDNA (COI) sequences estimated in Phyml. Localities of specimens are indicated after the voucher code. Numbers below branches indicate bootstrap support values >50. Note that the length of the branch leading to the outgroup is not to scale.

All specimens in mitochondrial Clade I (Fig. 2) and a series of specimens from Clade II were macerated in 10% KOH (aqueous solution) and stained with chlorazol black, and examined in 70% ethanol under a Leitz Wetzlar stereomicroscope to check for potentially diagnostic characters.

## Results

The North American “*tullia*” samples formed a well-supported monophyletic group (99% bootstrap proportion), sister to a clade comprising the European *tullia*, *C. rhodopensis,* and *C. amaryllis* (Table S1).

A maximum likelihood phylogeny of the North American “*tullia*” with a European *tullia* as outgroup revealed two well-supported clades (Fig. 2). The first clade (Clade I) comprised seven individuals grouped under three haplotypes, whereas the second (Clade II) included the remaining 42 individuals under 23 haplotypes. Relationships among haplotypes within the two clades were generally poorly resolved. All individuals except one in Clade I were from the Peace River region; the exception being a sample from Athabasca, more than 300 km from the former. The total number of segregating sites among the North American sequences was 67 (4.5%). The mean per cent sequence divergence between the two clades was 3%. The Ф_ST_ (a measure of genetic differentiation between groups, ranging from 0 to 1) between the clades was 89.67% and significantly different from zero (*P* = 0). The haplotype diversity (the probability that two randomly selected sequences in the sample are different; Nei 1987) and nucleotide diversity (the probability that two randomly chosen homologous nucleotide sites are different; Tajima 1983) of the North American samples were 94.64% (±1.69%) and 0.98% (±0.49%), respectively.

The high divergence between the two clades suggested the possibility of two different species. The male genitalia of individuals from the two clades were therefore compared to detect possible morphological differences. However, no potential taxonomically diagnostic characters were found. Only minimal individual differences in the setae on the uncus were observed, and these did not correspond to the haplotype groups.

**Figure 3:**
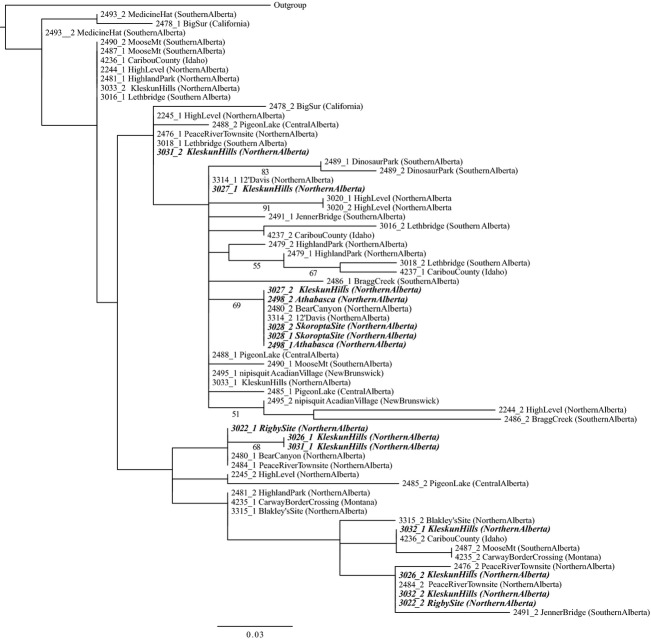
Maximum likelihood phylogeny of the nDNA (RpS5) sequences estimated in Phyml. Numbers below branches indicate bootstrap support values >50. Specimens that were part of Clade I in the mtDNA phylogeny are in bold italics.

We sequenced a 617-bp-long nuclear gene region – RpS5 (ribosomal protein subunit 5) to corroborate the surprisingly deep division. Although not often used in phylogeographic studies (but see Simonsen and Huemer 2014), RpS5 is known to exhibit high variation in nymphalid butterflies (Wahlberg and Wheat 2008). This gene was sequenced from 34 samples, including one European *tullia* sample used as outgroup. A total of 34 segregating sites (5.5%) comprising 36 unique haplotype sequences were found in the 33 North American samples, with a haplotype diversity of 96.55% (±0.98%) and nucleotide diversity of 0.63% (±0.36%). The maximum likelihood phylogeny of these haplotypes was poorly resolved and generally weakly supported (Fig. 3), with neither Clade I nor Clade II being represented.

**Figure 4:**
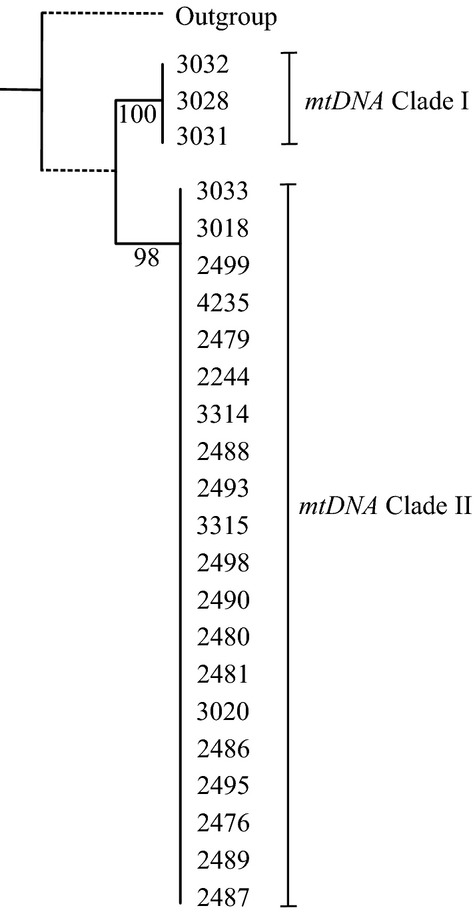
Maximum likelihood phylogeny of the combined *Wolbachia* gene dataset. Numbers below branches indicate bootstrap support values >50. The two mtDNA clades are infected by respective *Wolbachia* clades (strains). The length of the outgroup branch is not to scale.

A positive PCR-based assay for the presence of *Wolbachia* was obtained in 21 of 36 (ca. 58%) tested samples, from both COI Clades I and II. Sequences for the three *Wolbachia-*specific genes (wsp -580 bp, gatB *–* 416 bp and ftsZ – 480 bp) had two haplotypes each. For all genes, the two groups of individuals with differing *Wolbachia* haplotypes corresponded to COI Clade I and II, except for specimen 2498 from Athabasca. The maximum likelihood phylogeny of the combined three-gene dataset for *Wolbachia* is shown in Figure 4.

## Discussion

Results from the mitochondrial DNA alone indicate a deep split between clades I and II, corroborating Bromilow and Sperling (2011) who included 45 “*C. tullia*” samples. This suggests very old separation of two clades. There was also some north–south structuring in Clade II, with four clades or clusters segregating based on the occurrence in northern Alberta or southern central Alberta. While the Athabasca specimen nested with Clade I represents a substantial geographic jump, “*C. tullia*” is not dependent on native grasslands, which creates a possibility for migration between Peace River and Athabasca along roadsides and other weedy areas. The fact that neither mitochondrial clade is completely geographically restricted indicates that geographic isolation *per se* is inadequate to explain this structure. In fact, we found that several specimens (for instance from Kleskun Hill in the Peace River region) caught at the same time and in the same habitat segregated between the two divergent clades. Furthermore, the grassland ecosystem in the Peace River region is separated by 300-400 km from similar habitats in Alberta and British Columbia, but “*C. tullia*” is distributed continuously across these regions (Bromilow and Sperling 2011).

Importantly, this mtDNA structure was not corroborated by nuclear data or morphology, both of which showed no signs of divergence between the clades, and thus indicate considerable and recent nuclear gene flow between the two groups delimited by mtDNA. We first consider the hypothesis that the two clades represent unique species. Incomplete lineage sorting in RpS5 may perhaps explain the lack of resolution. However, the proportion of segregating sites in both genes is comparable (COI – 4.5%; RpS5 – 5.5%). If, as the COI tree suggests, strong and long-term reproductive isolation did exist between two clades, the RpS5 data should reflect this divergence despite the fact that nuclear genes have a fourfold greater effective population size. Morphological characters often evolve slowly in relation to genetic data, resulting in “cryptic” species (but see Joyce et al. 2009). This is evident in European *Coenonympha* where Higgins (1975) reported very little variation in a male genitalia survey; he remarked that amongst *Coenonympha,* “*C. oedippus* alone of European species has well marked specific characters in the genitalia, which otherwise are almost similar throughout the genus and of little value in species identification” (Higgins 1975; p. 267). However, even if this slow rate of morphological evolution of genitalia is considered, the male genitalia of the examined specimens were remarkably similar; minimal differences in the density of setae on the uncus were observed among specimens, and neither of these corresponded to the groups in the COI tree or geographical distributions. Setae on the uncus are generally very sparse in *Coenonympha* with the exception of the aforementioned *C. oedippus* (e.g., Higgins 1975; figs 380–394), and the difference reported here is in line with this. The setae were always small and thin, and their number varied between 6 and 12. We therefore conclude that the presence of two unique reproductively isolated species is supported by neither nuclear DNA nor morphology. Nonetheless, the lack of pattern in these characters does not *per se* contradict reproductive isolation.

The mitochondrial genome reflects female history, which may or may not be congruent with the phylogeographic history of the nuclear genome. Male-biased dispersal, where females are highly sedentary, can result in the kind of differences found in this study (Galtier et al. 2009). However, the Peace River samples are part of both clades, and it is hence implausible that restricted mitochondrial gene flow between the two clades is entirely due to the sedentary nature of females.

The best explanation for the observed mito-nuclear divergence is indirect selection on the mitochondrial genome as a result of disequilibrium with two *Wolbachia* strains. The strong divergence between the *Wolbachia* clades is confirmed by all three *Wolbachia* genes independently, indicating that the clades represent two different strains. The mitochondrial clades clearly show disproportionate association with these strains. The ecology of “*C. tullia*” is well known, and to our knowledge, there is no evidence for a sex ratio biased in favor of females. Cytoplasmic incompatibility, on the other hand, is common (Hurst and Jiggins 2005) and is usually unnoticed unless tested experimentally. All three phenotypes – male-killing, feminization, and cytoplasmic incompatibility – can lead to mitotypes being fixed (Hurst and Jiggins 2005). It seems likely that two such *Wolbachia* sweeps have given rise to the mitochondrial structure in “*C. tullia*”. It is also possible that mitochondrial haplotypes are retained from old extinctions due to selection by *Wolbachia* (Dyer et al. 2011). Intriguingly, one specimen (2498) did not follow this pattern as its COI sequence places it in Clade I, but its *Wolbachia* strain is typical of Clade II. While this does not alter the overall conclusion that different *Wolbachia* strains are responsible for the observed COI divergence, it does indicate that mtDNA transfer between lines infected with different *Wolbachia* strains is possible, if rare, between the two clades. This phenomenon deserves closer attention in future studies on *Wolbachia*-mediated genetic isolation. Interestingly, not all individuals tested positive for the presence of the bacterium. However, PCR-based assays do not detect the bacterium in all cases (Jeyaprakash and Hoy 2000). Moreover, our testing was performed based on leg tissue, and detection rates are known to vary among different kinds of tissue (Dobson et al. 1999). As transmission efficiency between mother and offspring is not always 100% (Hurst and Majerus 1993; Turelli and Hoffmann 1996), it is also possible that the infection rate has declined recently.

Cytoplasmic incompatibility between two strains is termed bidirectional incompatibility and manifests itself in the form of incompatible crosses between individuals harbouring different strains (Werren 1997). If both strains were ubiquitous in their respective clades, bidirectional incompatibility leads to complete reproductive isolation. Given sufficient time, this will result in complete lineage sorting even in nuclear genes. It is evident, however, that this has not happened in the case of “*C. tullia*”. Therefore, we surmise that one of the *Wolbachia* sweeps occurred very recently. It is even possible that *Wolbachia* may accelerate the divergence of mtDNA lineages, by competing with mitochondria for various amino acids during translation (Wu et al. 2004; Dunning Hotopp et al. 2006). The Canadian “*tullia*” populations studied here represent an excellent system to understand how *Wolbachia* prevalence evolves spatially and temporally and what effects these changes have on host population structure over time. Laboratory experiments to test the extent of cytoplasmic incompatibility (or other *Wolbachia* effects) will also be illuminating.

### Implications for mtDNA barcoding and phylogeography

The reliance on mitochondrial sequences alone for barcoding and phylogeography has received strong criticism in the context of infection by endosymbionts like *Wolbachia* (Hurst and Jiggins 2005; Gerth et al. 2011; Kvie et al. 2013), but continues unabated. If we had depended solely on COI data, the logical conclusion would have been that Clades I and II have been reproductively isolated for hundreds of thousands or even millions of years and represent two unique cryptic species. Mitochondrial sequences continue to be popular because of several advantages such as (1) ease of amplification due to high copy number per cell (2) their haploid nature makes identification of haplotypes straightforward, and (3) generally higher mutation rates compared with nuclear sequences. Preferably, variable nuclear markers such as microsatellites or AFLP should be used in conjunction with mtDNA sequences. However, the time and cost involved in developing or standardizing such markers is often prohibitive for routine use in phylogeographic studies. In such cases, nuclear sequences from quickly evolving genes can be used to corroborate mitochondrial structure (e.g., current study; Rokas et al. 2003). Next-generation sequencing technology holds promise for the future, as data from such techniques (e.g., RAD tags) typically span the entire genome.

*Wolbachia* are extremely common in insects – they are estimated to occur in up to 70% of all insects (Jeyaprakash and Hoy 2000; Duron et al. 2008). *Cardinium*, another cytoplasmic incompatibility inducing bacterium is estimated to infect ca. 7% of arthropods (Weeks et al. 2003). There may be several other parasites that have similar effects, and it is not realistic to detect all of these using PCR-based methods (Hurst and Jiggins 2005). Nevertheless, it is prudent to routinely check for *Wolbachia* whenever mtDNA is used for studies on groups known to be infected. Even in the absence of *Wolbachia*, a multimarker approach is highly recommended (Dupuis et al. 2012).

## Summary and conclusions

Two deeply divergent mitochondrial clades occurred among the “*C. tullia*” samples studied here. This divergence was not reflected in morphological differences or nuclear gene data. We found that the two mitochondrial clades are associated with respective *Wolbachia* strains and conclude that the most likely reason for their mito-nuclear divergence is that infection by the two *Wolbachia* strains resulted in strong, directional selection on the mitochondrial genome as they spread across populations, ultimately leading to fixation of two mitotypes. Our results further highlight the problems of sole reliance on mitochondrial barcodes for species delimitation and identification. It is imperative to corroborate such mitochondrial results with nuclear gene data in phylogeographic and population genetic studies on invertebrates.

## Supporting Information

**Table S1.** Maximum Likelihood phylogeny of the North American *Coenonympha* “*tullia*” samples used in this study along with five *C. tullia* from Europe and *Coenonympha* sequences from Kodandaramaiah and Wahlberg (2009; Systematic Entomology 34: 315–323). Numbers above branches indicate bootstrap support values greater than 50%.

**Figure S1.** Genbank accession numbers of samples used in the study along with details about their origin and collection.
