# Supplementary figures and images for: Deceptive single-locus taxonomy and phylogeography: Wolbachia-associated divergence in mitochondrial DNA is not reflected in morphology and nuclear markers in a butterfly species
Source: Ecol Evol. 2013 Nov 25;3(16):5167–76. doi: 10.1002/ece3.886 (PMC3892326; doi:10.1002/ece3.886)

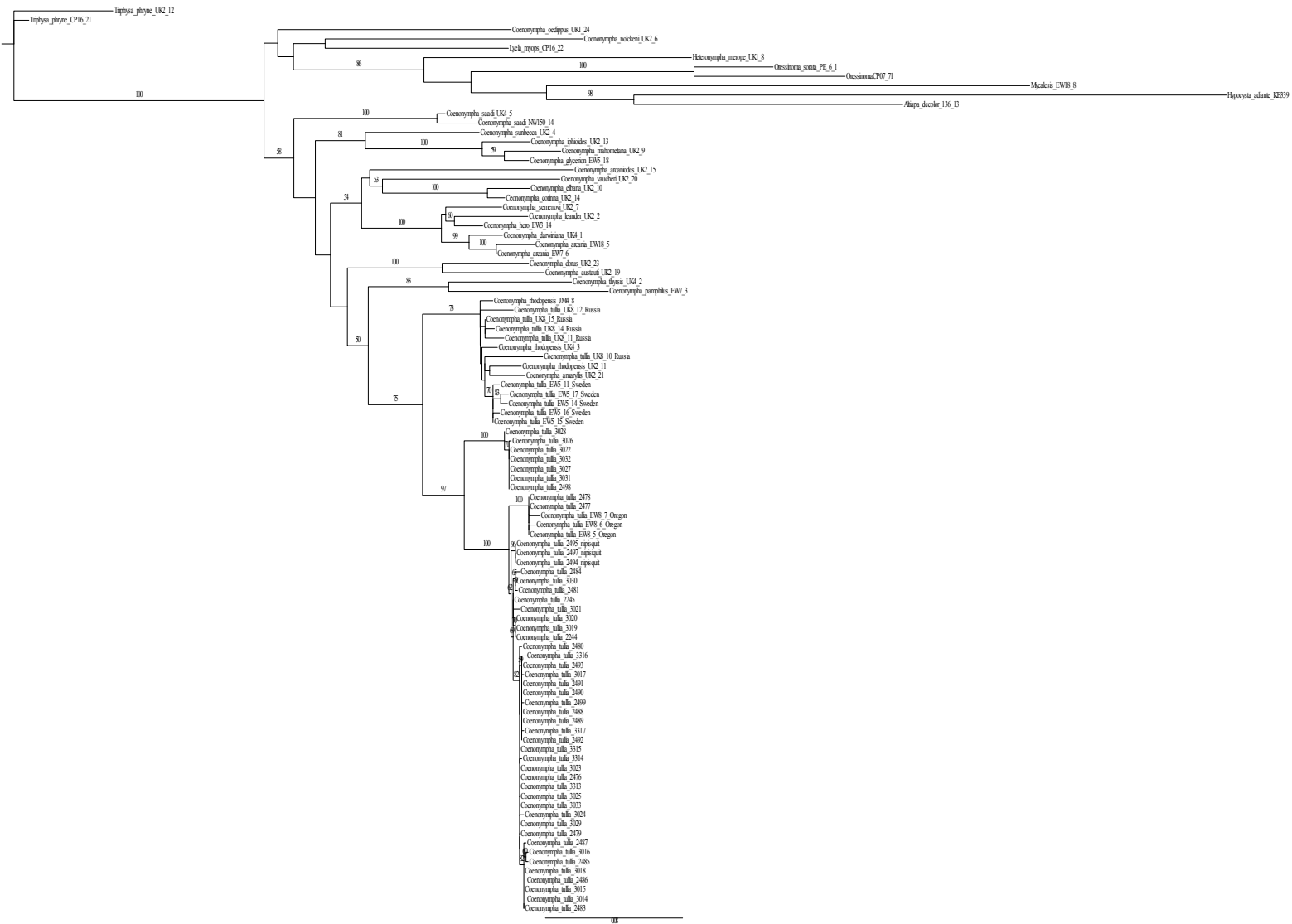

Supplement: Supplementary file 2 [file ece30003-5167-SD2.pdf]
